# A novel autosomal dominant *GREB1L* variant associated with non-syndromic hearing impairment in Ghana

**DOI:** 10.1186/s12920-022-01391-w

**Published:** 2022-11-10

**Authors:** Samuel Mawuli Adadey, Elvis Twumasi Aboagye, Kevin Esoh, Anushree Acharya, Thashi Bharadwaj, Nicole S. Lin, Lucas Amenga-Etego, Gordon A. Awandare, Isabelle Schrauwen, Suzanne M. Leal, Ambroise Wonkam

**Affiliations:** 1 West African Centre for Cell Biology of Infectious Pathogens (WACCBIP), University of Ghana, LG 54, Accra, Ghana; 2 Division of Human Genetics, Faculty of Health Sciences, University of Cape Town, Cape Town, 7925 South Africa; 3 Department of Neurology, Center for Statistical Genetics, Sergievsky Center, Columbia University Medical Centre, New York, NY 10032 USA; 4 Taub Institute for Alzheimer’s Disease and the Aging Brain, Columbia University Medical Centre, New York, NY 10032 USA; 5 Department of Genetic Medicine, McKusick-Nathans Institute, Johns Hopkins University School of Medicine, Baltimore, MD 21205 USA

**Keywords:** Hearing impairment, *GREB1L*, Ghana

## Abstract

**Background:**

Childhood hearing impairment (HI) is genetically heterogeneous with many implicated genes, however, only a few of these genes are reported in African populations.

**Methods:**

This study used exome and Sanger sequencing to resolve the possible genetic cause of non-syndromic HI in a Ghanaian family.

**Results:**

We identified a novel variant c.3041G > A: p.(Gly1014Glu) in *GREB1L* (DFNA80) in the index case. The *GREB1L*: p.(Gly1014Glu) variant had a CADD score of 26.5 and was absent from human genomic databases such as TopMed and gnomAD. *In silico* homology protein modeling approaches displayed major structural differences between the wildtype and mutant proteins. Additionally, the variant was predicted to probably affect the secondary protein structure that may impact its function. Publicly available expression data shows a higher expression of *Greb1L* in the inner ear of mice during development and a reduced expression in adulthood, underscoring its importance in the development of the inner ear structures.

**Conclusion:**

This report on an African individual supports the association of *GREB1L* variant with non-syndromic HI and extended the evidence of the implication of *GREB1L* variants in HI in diverse populations.

**Supplementary Information:**

The online version contains supplementary material available at 10.1186/s12920-022-01391-w.

## Background

Globally, over 124 genes have been implicated in hearing impairment (HI) [1] with connexins, especially *GJB2*, accounting for over 50% of autosomal recessive cases [2, 3]. A recent meta-analysis of HI genes from Africa identified 46 HI genes from 17 African countries confirming the heterogeneity of HI [4]. The most frequently associated genes with HI from Africa were *GJB2*, *MYO15A*, and *ATP6V1B1*. However, the contribution of connexins to the HI in sub-Saharan Africa is negligible except for a few cases from some countries including Ghana [5–7]. In Ghana, *GJB2*:p.(Arg143Trp) was reported as a founder variant [8] and recent studies have shown that the variant accounts for about 25–42% of familial cases [7, 9].

Growth Regulation by Estrogen in Breast cancer 1 Like gene (*GREB1L)*, a pre-migratory neural crest regulator, plays a vital role in early metanephros and genital development [10, 11]. Wild-type *GREB1L* functions in retinoic acid receptor (RAR) gene co-activation and the development of vestibulocochlear, renal system, genital tract, and ventricular tract [12]. Variations in *GREB1L* have been implicated in renal hypodysplasia (aplasia 3) (RHDA3), Autosomal Dominant hearing impairment (DFNA80), and Mayer–Rokitansky–Küster–Hauser (MRKH) syndrome [11–13] Variations in *GREB1L* implicated in HI have been shown to be either autosomal dominantly inherited (with or without reduced penetrance) or de novo [11, 14, 15]. Patients with HI due to variant in *GREB1L* typically display bilateral profound non-syndromic (NS) HI. Additionally, when imaging data are available cochleovestibular abnormalities are observed [11]. When performed, a normal renal ultrasound and urine analysis were observed in investigated NSHI probands with *GREB1L* variants [14].

In this study, we present a novel case with a heterozygous *GREB1L* p.(Gly1014Glu) variant associated with congenital profound NSHI in a Ghanaian family. Thus, describing the ninth family with a novel variant contributing to the expansion of the genetic and demographic spectrum of *GREB1L* pathogenic variants.

## Materials and methods

### Study participants

**Fig. 1:**
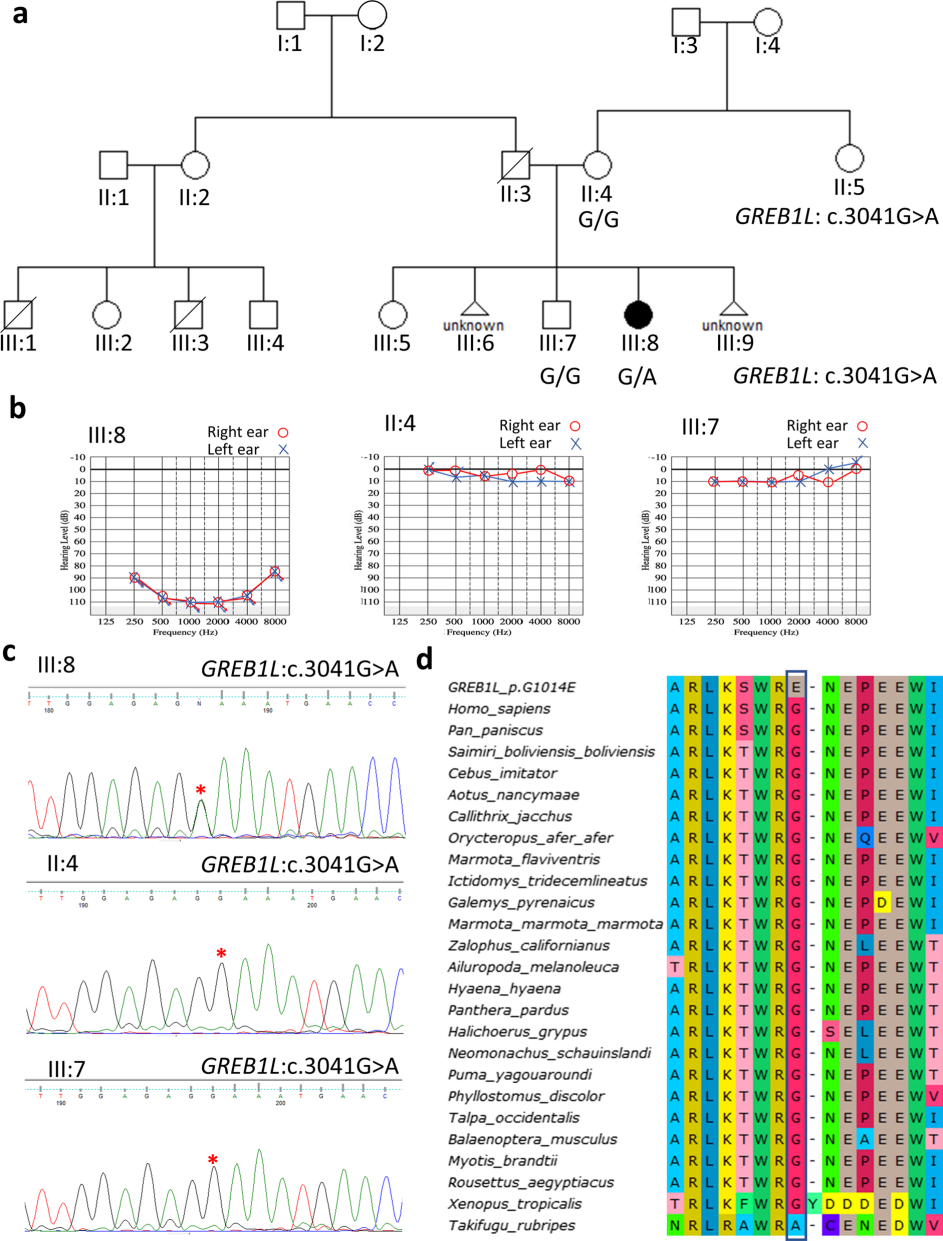
Pedigree, hearing impairment phenotype, and Sanger sequence validation of *GREB1L*: c.3041G > A variant. **A** Pedigree and genotype of the affected child (III:8), her mother (II) and brother. Squares represent males and circles females. The individual with the filed symbol is affected and clear symbols represent individuals without hearing impairment. **B** Pure tone audiograms of affected and unaffected family members. **C** Chromatograms showing *GREB1L*: c.3041G > A genotypes. **D** GREB1L protein sequence alignment has shown conservation of the amino acid at position 1014

The family with an affected proband was part of a large study aimed at investigating the association of genetic markers to congenital hearing impairment among patients at the schools for the Deaf in Ghana. The affected family member was screened and negative for *GJB2* pathogenic variants [7]. In combination with a structured questionnaire, the medical records of the proband were evaluated to rule out any potential environmental or acquired cause of HI. The proband was examined by a medical geneticist and an ear, nose, and throat specialist. Urine analysis was conducted for the proband using Urine Test Paper Strips. To assess the hearing thresholds, pure-tone audiometric examination was performed using KUDUwave™ Audiometer-eMoyo Technologies (Johannesburg, Gauteng, South Africa) (Fig. 1). Genomic DNA (gDNA) was extracted from venous blood obtained from 3 family members [(II:4, III:7, and III:9) (Fig. 1)] QIAamp DNA Blood Maxi Kit^®^ (Qiagen, USA).

### Exome sequencing

Whole exome sequencing (WES) was performed on the gDNA obtained from the affected proband. Briefly, Quantus Fluorometer (Promega, Madison, WI) was used to assess the quality of the gDNA prior to the exome sequencing. Exome libraries were prepared using SureSelect V4 + UTR 71 Mb All Exon Capture Kit (Agilent Technologies, Inc., Santa Clara, CA, USA), ~ 3–5 µg of the DNA was fragmented with ultrasound using a Covaris^®^ instrument (Covaris, Inc., Woburn, MA, USA). Sequencing of the libraries was performed on Illumina HiSeq 2000 (Illumina, San Diego, CA) to produce paired-end reads of 100 bp. The Illumina BaseSpace app suite was used for exome sequencing mapping and variant calling. The sequence reads were aligned to the human reference genome (hg19/GRCh37) using Illumina DRAGEN Germline Pipeline version 05.021.408.3.4.12. Reads were sorted and marking of duplicates was performed using Picard. The Genome Analysis Toolkit (GATKv4.1.7) software package [16] was used to conduct joint variant calling for single nucleotide variations (SNV) and Insertion/Deletions (Indels). The sex of the family member that underwent WES was verified using plink (version 1.9) [17, 18]. Last, copy number variants (CNV) were called using the copy number inference from exome reads algorithm (CoNIFER) [19].

### Annotation and filtering

We performed annotation and filtering using an in-house pipeline built on ANNOVAR as described previously [20]. After checking known pathogenic variants for NSHI regardless of their frequency, filtering of SNVs and indels was performed using Genome Aggregation Database (gnomAD) [21] with a population-specific minor allele frequency of < 0.005 [for homozygous and potentially compound heterozygous variants and variants on the X chromosome AR] and < 0.0005 for heterozygous variants. Synonymous and intronic variants that were not close to a splice site region were removed. Variants that met the above criteria were further prioritized based on *in silico* prediction scores from Sorting Intolerant From Tolerant (SIFT); MutationTaster; combined annotation dependent depletion (CADD); Genomic Evolutionary Rate Profiling (GERP++); polymorphism phenotyping v2 (PolyPhen-2); and deleterious annotation of genetic variants using neural networks (DANN). The variants were further assessed with information from Hereditary Hearing Loss Homepage (HHL), Online Mendelian Inheritance in Man (OMIM), Human Phenotype Ontology (HPO), and ClinVar databases. Allele frequencies were also assessed using the TOPMed Bravo database. The American College of Medical Genetics and Genomics and Association (ACMG-AMP) guidelines for HI [22] were followed to evaluate clinical significance.

CNVs were annotated and filtered with AnnotSV [23] and an in-house pipeline that interrogates BioMart [24] and the Database of Genomic Variants [25].

### Sanger sequence validation of the candidate variant from WES

To verify segregation of the *GREB1L*: c.3041G > A: p.(Gly1014Glu) candidate variant, Sanger sequencing was performed for all the family members from whom a DNA sample was obtained. Allele specific primers (Forward: AAACTACAGCCCTCGTTCCT, Reverse: CCTTGAGGGGTGCAGGAATAG) were used to PCR amplify the region of the *GREB1L* gene containing the variant. The PCR amplicons were cleaned using exonuclease 1 and alkaline phosphatase after which they were sequenced using BigDye™ Terminator v3.1 Cycle Sequencing Kit. The sequencing products were resolved and analyzed by ABI 3130XL Genetic Analyzer® (Applied Biosystems, Foster City, CA, USA). The Sanger sequence data files were analyzed using FinchTV v1.4.0, and UGENE v34.0.

### GREB1L protein modeling

The 3D structure of GREB1L [1923 amino acids (aa) long] predicted by the highly accurate AlphaFold method was retrieved as a PDB file from the AlphaFold protein structure database and used as a template for the modeling of the mutant [GREB1L: c.3041G > A: p.(Gly1014Glu)] structure using SwissModel. Since the downstream structure refinement by GalaxyWeb [26] required the structure to be ≤ 1000aa, the protein was truncated by removing 600aa from the N-terminal and 369aa from the C-terminal to retain a 954aa sequence that contained the mutant site. The truncation was further deemed to be desirable since there were no apparent interactions between the variant site and distant residues in the wildtype AlphaFold-predicted structure. In addition, the wildtype glycine residue (G^1014^) resides within a region in the protein that was predicted with high confidence by AlphaFold. The refined mutant truncated structure and the full-length wildtype structure were then analyzed using PyMol [27]. The truncated wildtype and mutant proteins were further analyzed on PROTTER [28] and PSIPRED v4.0 programs [29] to determine the location and predictive effect of the variant on secondary structure formation.

### **Expression of*Greb1l*in mouse inner ear**

To examined the expression of *GREB1* in the mouse inner ear, we obtained and analyzed single cell RNA-seq data at different developmental stages from a publicly available database, gene Expression Analysis Resource (gEAR) suite [30]. The gEAR suite is a data deposition, display, analysis, and interrogation database which consist of expression data of different organisms such as mouse, human, rat, and zebrafish [30].

## Results

### Clinical evaluation

No known syndrome was identified in the proband when examined by a medical geneticist. At the time of sample collection, the affected participant did not have any clinical signs of renal/kidney dysfunction and no abnormal urine analysis parameter was observed. The affected individual (III:8, Fig. 1) presented with profound bilateral, symmetrical sensorineural NSHI while the unaffected family members had normal hearing (Fig. 1b). We were unable to perform any imaging to assess inner ear malformations which may lay at the basis of the NSHI in the proband.

### ***GREB1L***: **c.3041G > A: p.(Gly1014Glu) identified through exome sequencing**

Whole exome sequence data was generated from gDNA samples from one affected (III:8) family member. The analysis of the exome data identified a mono-allelic *GREB1L* variant (NM_ 001142966.2: c.3041G > A) in the affected individual (Table 1). The variant was predicted as variant of uncertain significance with some pathogenic evidence based on the ACMG guidelines [31] and Varsome [32], and it was predicted to be pathogenic by different bioinformatic predictive tools including SIFT, PolyPhen, and FATHMM (Additional file 1: Table S1). In addition, the variant is absent from the Deafness Variation Database (DVD), gnomAD and TopMed databases and its position is conserved amongst species (phyloP100way = 5.3). Family members with normal hearing were homozygote wildtype (G/G) (Fig. 1a). The identified missense c.3041G > A variant was confirmed by Sanger sequencing (Fig. 1c). No relevant CNVs were identified.

**Table 1:** *GREB1L* variants associated with HI

Family type	cDNA change*	amino acid change	Mode of inheritance	CADD score (v1.6)	TopMed	gnomAD v3.1.2	Hearing loss	Inner ear imaging phenotype	Country	Reference
Isolated	c.347 C > T (rs1333304296)	p.(Thr116Ile)	AD**	27.2	Absent	Absent	Profound bilateral SNHI	Bilateral cochlear aplasia, bilateral dysplastic vestibule and semicircular canals; bilateral cochlear nerve aplasia	Egypt	[14]
Familial	c.848 A > G	p.(Asn283Ser)	AD	16.2	Absent	Absent	Profound bilateral SNHI	NA	Pakistan	[14]
Isolated	c.982 C > T (rs1555648043)	p.(Arg328*)	de novo/ AD**	36.0	Absent	Absent	Profound bilateral SNHI	Case 1: Bilateral cochlear aplasia, bilateral dysplastic vestibule and semicircular canals; bilateral cochlear nerve aplasia. Case 2: Cochlear hypoplasia type 1 (right): Cochlear aplasia with dilated vestibule (left)	US, Korea	[11] [15]
Isolated	c.1079T > A	p.(Leu360*)	AD**	36.0	Absent	Absent	Profound bilateral HI	Cochlear aplasia with dilated vestibule (right): bilateral common cavity and Cochlear aplasia with dilated vestibule (left)	Korea	[15]
Isolated	c.3041G > A	p.(Gly1014Glu)	AD or de novo	26.5	Absent	Absent	Profound bilateral SNHI	NA	Ghana	This study
Isolated	c.4368G > T	p.(Glu1410fs)	de novo	36.0	Absent	Absent	Profound bilateral SNHI	Unilateral cochlear aplasia, unilateral incomplete partition type I, bilateral dysplastic vestibule and semicircular canals; bilateral cochlear nerve aplasia	US	[11]
Isolated	c.5618T > C	p.(Leu1873Pro)	AD**	28.9	Absent	Absent	Profound bilateral HI	Bilateral common cavity	Korea	[15]

*AD*  Autosomal dominantly inherited;* NA* Not available

*Based on NM_001142966.2. **Reduced penetrance seen in family.

### ***In silico*GREB1L protein analysis**

**Fig. 2:**
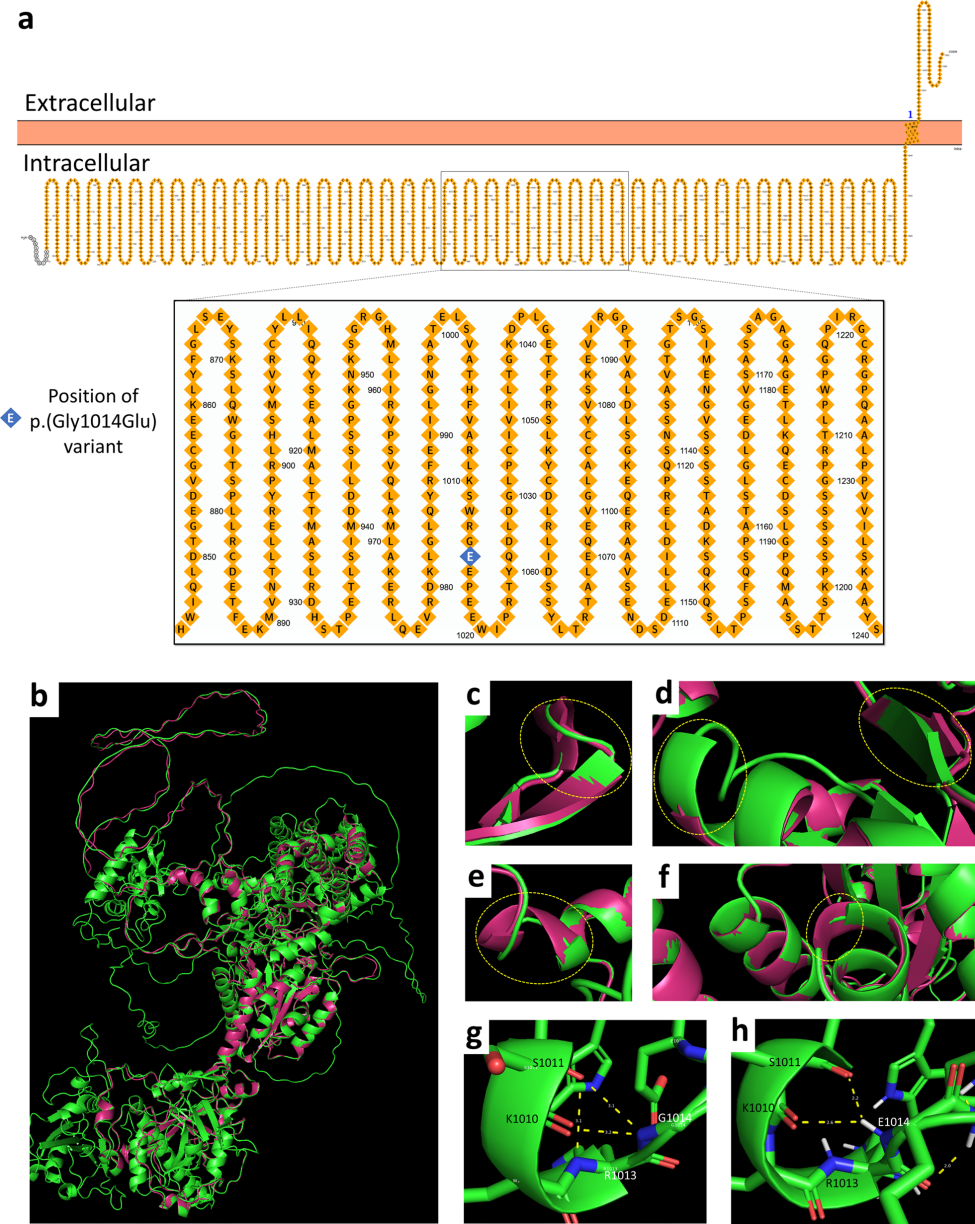
*In silico* GREB1L protein analysis showing structural variations between the modelled wildtype and mutant proteins. **A** Cellular organization of GREB1L as predicted using PROTTER [28], an online bioinformatic tool. Homology modelling represented in **B** Superimposition of full-length wildtype and truncated mutant GREB1L structures. **C** Formation of helix at ^1469^SSMLG^1473^ in the mutant. **D** shortening of helix at ^1559^KY^1560^ and beta strand at ^1544^LHLLVV^1549^ in the mutant. **E** and **F** Extension of helices at ^1093^DLSG^1096^ and ^923^TT^924^ in the mutant. Yellow circles denote sites of different secondary structural changes of the mutant compared to the wildtype structures. Hydrogen-bond formation of **G** wildtype and **H** mutant residues with nearby residues. H-bond between K^1010^ and R^1013^ residues is lost in the mutant, and the H-bond lengths are shortened in the mutant compared to the wildtype

Protein sequence alignment was conducted to study the evolutionary conservation of amino acid position 1014 of the GREB1L protein (Fig. 1d). The position 1014 was found in the intracellular domain of the protein (Fig. 2a), and glycine (G) was conserved at this position for all the species studied except for *Xenopus tropicallis* and *Takifugu rubripes* (Fig. 1d) which are evolutionarily distant from mammals. It is however worth mentioning that the amino acid at this position was conserved in the mammalian species studied, suggesting its importance to the structure of GREB1L protein. The change from a neutral amino acid, glycine, to a negatively charged glutamate (E) at this position likely affected the protein structure and function. Analysis of the protein models showed that there was an excellent superimposition between the full-length wildtype structure and the mutant structure (Fig. 2b). Comparison of the superimposed structures revealed several secondary structural changes among the wildtype and mutant structures including: the gain of a short helix in the mutant structure in a region that formed a loop in the wildtype structure between residues serine-1469 and glycine-1474 (i.e., ^1469^SSMLG^1474^) (Fig. 2c), a shortening of a helix around residues leucine − 1559 and tyrosine-1560 (i.e., ^1559^KY^1560^) and a shortening of a beta strand between residues leucine-1544 and valine-1549 (i.e., ^1544^LHLLVV^1549^) in the mutant (Fig. 2d), the extension of a helix between residues aspartate-1093 and glycine-1096 (i.e., ^1093^DLSG^1096^) and between threonine-923 and threonine-924 (i.e., ^923^TT^924^) in the mutant (Fig. 2e and f). PSIPRED, a bioinformatic tool, also predicted that the E1014 residue in the mutant forms a helix which is absent in the wildtype (blue rectangles in Additional file 1: Fig. S1). Other major secondary structural changes were found when the wildtype was compared to the mutant (red rectangles in Additional file 1: Fig. S1).

The wildtype G^1014^ residue is predicted by AlphaFold to form two hydrogen (H-) bonds with lysine-1010 (K^1010^) and serine-1011 (S^1011^), while K^1010^ forms another H-bond with arginine-1013 (R^1013^) (Fig. 2g). Interestingly, although the H-bonds formed with the K^1010^ and S^1011^ residues are retained in the mutant structure, the H-bond bond between the K^1010^ and R^1013^ residues is lost (Fig. 2h). In addition, the H-bonds formed by the mutant E^1014^ residue appear to be shorter (hence stronger) than those formed by the wildtype residue. Therefore, it is probable that the mutant residue imposes a change in the geometry of nearby residues, particularly the Arginine residue. Indeed, the mutant E^1014^ residue is larger than the wildtype G^1014^ residue in addition to being polar charged.

### **Expression of*Greb1l*in mouse inner ear**

Our study further explored single cell RNA-seq (scRNA-seq) data from gEAR to study Greb1l expression in the developing inner ear [30], as scRNA-seq data had not been interrogated in previous studies. The scRNA-seq data covered expression of Greb1l in spiral, glia, and hair cells obtained at six developmental stages (E15.5, P1, P8, P12, P14, and P30). Greb1l expression was the most prominent in the developing spiral ganglion, where it was upregulated at the E15.5 developmental stage decreased over the developmental stages (Additional file 1: Fig. S2). In the glia and hair cells, it was up regulated at P8 and P12 developmental stages respectively (Additional file 1: Fig. S2). The Greb1l expression data supports its critical role in the development and functioning of the inner ear and cochlear nerve.

## Discussion

Using WES, we identified a previously unreported variant in *GREB1L* [c.3041G > A: p.(Gly1014Glu)], associated with the NSHI phenotype in an individual of African ancestry from Ghana, and a wider investigation in other African countries and diaspora is needed. This finding presents the first report of a *GREB1L* variant association with HI from sub-Saharan Africa. The identified heterozygous missense p.(Gly1014Glu) variant was predicted as a possible loss of function mutation that suggests haploinsufficiency as the pathological basis for the associated phenotype, and this is consistent with earlier *GREB1L* reports [11, 33].

Variants in *GREB1L* were associated with HI in individuals from the US, Egyptian, Korean, and Pakistani populations [11, 14, 15]. Two de novo* GREB1L* pathogenic variants; p.(Glu1410fs) and p.(Arg328*) were associated with profound HI, cochlear aplasia, incomplete partition type I (IP-I) and cochleovestibular nerve malformations [11]. An inherited missense variant; p.(Asn283Ser) was found to segregate with HI in a Pakistani family with 3 members having profound bilateral NSHI [14]. Similarly, a missense variant [p.(Thr116Ile)] in the gene was reported in NSHI with bilateral cochlear aplasia and cochlear nerve aplasia in Egyptian family [14]. Recently, three additional Korean cases with severe inner ear malformations were reported with rare heterozygous *GREB1L* variants [p.(Arg328*), p.(Leu360*), p.(Leu1873Pro)] [15] (Table 1). Most of the *GREB1L* variants associated with HI (6/7) were in the intracellular domain of the protein and only one variant was found in the extracellular domain. The extracellular domain variant [p.(Leu1873Pro)] was associated with a less severe inner ear malformation (Table 1; Additional file 1: Fig. S3). The increased severity of inner ear malformations observed in patients with intracellular domain variants may be due to the reduction or disruption of the protein’s gene regulatory activity which is associated with this domain [34].

*GREB1L* (OMIM: 617,782) is located on 18q11.1-q11.2 of the human genome and has been implicated in Autosomal Dominant deafness and renal hypodysplasia/aplasia. Although the precise function of *GREB1L* remains uncertain, its involvement in the neural crest suggests its associated disorders are neurocristopathies [35]. *GREB1L* was predicted to be involved in retinoic acid signaling based on its similarity with *GREB1* [12]. The molecular mechanism of HI pathogenesis of *GREB1L* remains unclear, however, *Greb1L* knockout mice were reported to develop severe craniofacial abnormalities and RNA-Seq data from laser capture micro-dissected (LCM) mouse tissues during craniofacial development shows that *Greb1l* was preferentially expressed at the early stages of mouse development [11, 36]. In zebrafish, greb1l has been implicated in Hoxb1 and Shha signaling, with critical role in pathways in the inner ear and cranial nerve development [33, 37, 38]. Inner ear imaging examination for the proband would have been relevant for the comprehensive description of the associated phenotype however, it was not conducted due to major challenges faced at the time of participant recruitment and sample collection. Yet, we believe it is important to report these cases in the African population, even though we are typically more limited in clinical phenotyping in these populations.

Similar to the gene’s involvement in multiple tissues development as seen in mice studies [39], pleiotropic effects are seen in humans. Several studies associated variants in *GREB1L* to congenital kidney malformations/agenesis [39–41], urogenital adysplasia, and Mayer-Rokitansky‐Kuster‐Hauser syndrome [13, 42] suggesting its role in the functioning of the urogenital systems. However, at the time of sample collection, the proband did not show any sign/symptoms of urogenital disorders. Furthermore, urine analysis showed no signs of kidney/urinary tract disorders in the proband.

Previous studies have shown that *GREB1L* pathogenic variants exhibits a maternal bias inheritance which may be explained by imprinting or low male fertility due to *GREB1L* variants [14, 39]. The mother of the index case was unaffected which does not favor the maternal bias observation from the previous reports. The variant p.(Gly1014Glu) identified in this study may be a de novo variant since it was absent in the unaffected mother and brother. Biological samples were not obtained from the deceased father of the affected child and hence his genotype is unknown. Nonetheless, the low rate of paternal inheritance of *GREB1L* variants [39, 40] and absence from gnomAD/TopMed supports our claim of the *GREB1L*: p.(Gly1014Glu) as a likely de novo variant.

## Conclusion

Using exome sequencing, we identified a variant in *GREB1L* [p.(Gly1014Glu)] as the possibly associated genetic cause of HI in a Ghanaian individual with profound HI. *In silico* techniques predicted the novel missense substitution as the likely cause of pathogenicity which led to the observed HI phenotype. This was evident in major structural difference observed between the wildtype and mutant GREB1L modelled protein, which is likely to affect the protein function. *GREB1L* variants should be investigated in other African populations and its inclusion in hearing panels should be considered.

## Supplementary Information

**Additional file 1: Fig. S1.** Secondary structure prediction of GREB1L protein. The effect of the variant on secondary structure formation was examined using PSIPRED [1], a bioinformatic tool. Predicted secondary structures for the (A) wildtype and (B) mutant proteins. Blue rectangles were used to indicate the absence and presence of a helix at the mutation site of the wildtype and mutant proteins respectively. Red rectangles were used to highlight the sites where differences were observed in the structures of the wildtype compared to the mutant.** Fig. S2.** Single cell RNA expression of Greb1l at different developmental stages in the mouse inner ear. The spiral ganglion (SGD), glia, and hair cell (HC) RNA-seq data sets were retrieved from gEAR [2].** Fig. S3.** A diagram mapping GREB1L variants to their associated protein domains.** Table S1.** In silico prediction of clinical significance/pathogenicity

## Data Availability

*GREB1L*: p.(Gly1014Glu) Sanger sequence generated from the proband was submitted to GenBank with the accession code ON390796. Data on *GREB1L*: p.(Gly1014Glu) variant has been added to dbSNP and will be publicly available when the next dbSNP Build (B156) is released (https://www.ncbi.nlm.nih.gov/snp/). All other relevant data supporting the key findings of this study are available within the article and its Supplementary Material. Due to lack of ethical approval, individual-level whole-exome sequence data cannot be made publicly available; however, it can be obtained from the corresponding author [A.W.] upon reasonable request.

## Abbreviations

HI: Hearing impairment
GREB1L: GREB1-like retinoic acid receptor coactivator
PLP: Pathogenic or likely pathogenic
CADD: Combined annotation dependent depletion
NSHI: Non-syndromic hearing impairment
MRKH: Mayer–Rokitansky–Küster–Hauser
WES: Whole exome sequencing
SNV: Single-nucleotide variant
AD: Autosomal dominant
GERP + +: Genomic evolutionary rate profiling
DANN: Deleterious annotation of genetic variants using neural networks
HHL: Hereditary hearing loss homepage
OMIM: Online mendelian inheritance in man
HPO: Human phenotype ontology
ACMG-AMP: American College of Medical Genetics and Genomics and Association for Molecular Pathology
GEO: Gene expression omnibus
SHIELD: Shared harvard inner-ear laboratory database
