# Supplementary material for: A novel autosomal dominant GREB1L variant associated with non-syndromic hearing impairment in Ghana
Source: BMC Med Genomics. 2022 Nov 10;15:237. doi: 10.1186/s12920-022-01391-w (PMC9648021; doi:10.1186/s12920-022-01391-w)
Supplement: Supplementary file 1 — Additional file 1: Fig. S1. Secondary structure prediction of GREB1L protein. The effect of the variant on secondary structure formation was examined using PSIPRED [1], a bioinformatic tool. Predicted secondary structures for the (A) wildtype and (B) mutant proteins. Blue rectangles were used to indicate the absence and presence of a helix at the mutation site of the wildtype and mutant proteins respectively. Red rectangles were used to highlight the sites where differences were observed in the structures of the wildtype compared to the mutant. Fig. S2. Single cell RNA expression of Greb1l at different developmental stages in the mouse inner ear. The spiral ganglion (SGD), glia, and hair cell (HC) RNA-seq data sets were retrieved from gEAR [2]. Fig. S3. A diagram mapping GREB1L variants to their associated protein domains. Table S1. In silico prediction of clinical significance/pathogenicity [file 12920_2022_1391_MOESM1_ESM.docx]

**Supplementary Material**


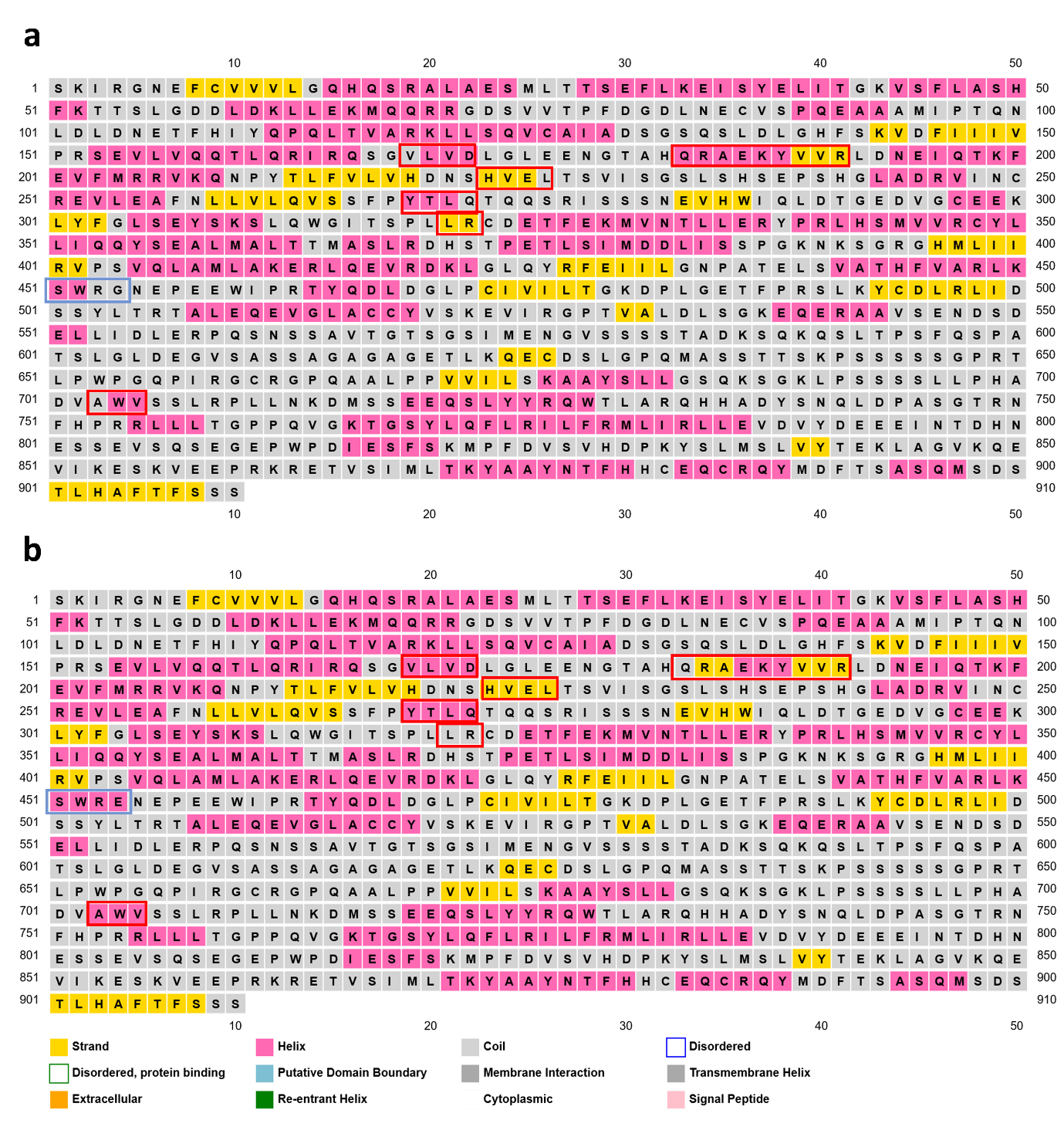


**Supplementary Figure 1: Secondary structure prediction of GREB1L protein.** The effect of the variant on secondary structure formation was examined using PSIPRED [1], a bioinformatic tool. Predicted secondary structures for the (A) wildtype and (B) mutant proteins. Blue rectangles were used to indicate the absence and presence of a helix at the mutation site of the wildtype and mutant proteins respectively. Red rectangles were used to highlight the sites where differences were observed in the structures of the wildtype compared to the mutant.


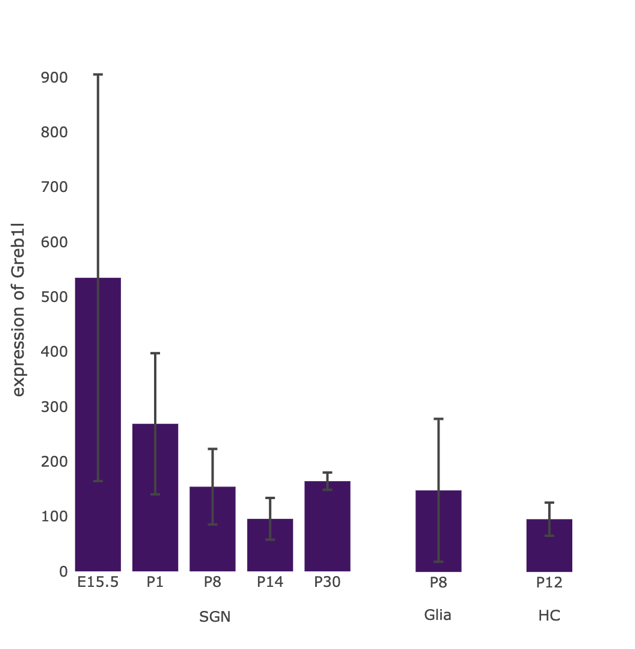


**Supplementary Figure 2: Single cell RNA expression of Greb1l at different developmental stages in the mouse inner ear.** The spiral ganglion (SGD), glia, and hair cell (HC) RNA-seq data sets were retrieved from gEAR [2].

**
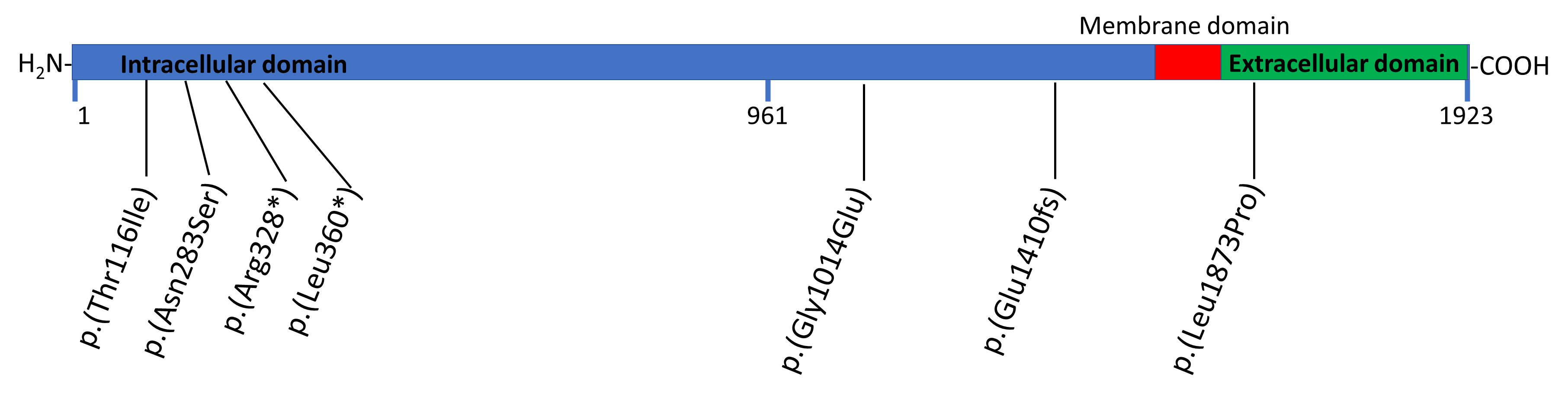
**

**Supplementary Figure 3: A diagram mapping GREB1L variants to their associated protein domains.**

**Supplementary Table 1: *In silico* prediction of clinical significance/pathogenicity**

| Variant database/browser or prediction tool | Evidence/Score | Prediction |
| --- | --- | --- |
| ACMG | PM2_supporting, PP3_supporting, PS2_moderate | Uncertain significance |
| SIFT | 0.024 | Damaging |
| PolyPhen | 0.82 | Damaging |
| PolyPhen-2 | 0.99 | Damaging |
| FATHMM-MKL | 0.9779 | Damaging |
| FATHMM-XF | 0.089 | Neutral |
| MutationTaster | 0.81 | Disease causing |
| EIGEN | 0.857 | Pathogenic |
| CADD score | 26.5 | Deleterious |
| MetaLR | 0.54 | Damaging |
| Revel | 0.36 | Benign |
| PrimateAI | 0.54 | Tolerated |
| PhyloP100way_vertebrate | 5.305 | Evolutionally conserved |
| GERP_RS | 5.42 | Conserved across multiple species |
| EIGEN | 0.74 | Pathogenic |

**Supplementary References**

1. Buchan DW, Jones DT: The PSIPRED protein analysis workbench: 20 years on. Nucleic acids research 2019, 47(W1):W402-W407.

2. Orvis J, Gottfried B, Kancherla J, Adkins RS, Song Y, Dror AA, Olley D, Rose K, Chrysostomou E, Kelly MC: gEAR: Gene Expression Analysis Resource portal for community-driven, multi-omic data exploration. Nature methods 2021, 18(8):843-844.
